# Para-probiotics for Preterm Neonates—The Next Frontier

**DOI:** 10.3390/nu10070871

**Published:** 2018-07-05

**Authors:** Girish Deshpande, Gayatri Athalye-Jape, Sanjay Patole

**Affiliations:** 1 Department of Neonatology, Nepean Hospital, Kingswood, NSW 2747, Australia; girish.deshpande@health.nsw.gov.au; 2 Sydney Medical School Nepean, University of Sydney, Kingswood, NSW 2747, Australia; 3 Department of Neonatology, King Edward Memorial Hospital for Women, Perth, WA 6008, Australia; gayatri.jape@health.wa.gov.au

**Keywords:** infant, newborn, probiotics, review, sepsis

## Abstract

Current evidence supports the use of probiotics in preterm neonates for prevention of necrotizing enterocolitis, mortality and late onset sepsis. Despite the strong evidence, the uptake of this intervention has not been universal due to concerns including probiotic sepsis, pro-inflammatory response and transmission of antibiotic resistance. Critically ill extremely preterm neonates with potentially compromised gut integrity are at higher risk of probiotic sepsis due to translocation. In most countries, probiotics are sold as food supplements with poor quality control. The traditional definition of probiotics as “live microorganisms” has been challenged as many experts have questioned the importance of viability in the context of the beneficial effects of probiotics. Paraprobiotics (ghost probiotics), are defined as non-viable microbial cells (intact or broken) or crude cell extracts (i.e., with complex chemical composition), which, when administered (orally or topically) in adequate amounts, confer a benefit on the human or animal consumer. Current evidence indicates that paraprobiotics could be safe alternatives to probiotics in preterm neonates. High-quality pre-clinical and clinical studies including adequately powered randomised controlled trials (RCTs) are warranted in preterm neonates to explore this new frontier.

## 1. Introduction

Despite the recent advances in technology and progress in research, necrotising enterocolitis (NEC), mortality, late onset sepsis (LOS), and long-term neurodevelopmental impairment (NDI) continue to remain a major health burden in preterm neonates [1,2]. NEC continues to be major gastrointestinal emergency in ~7% of preterm very low birth weight (VLBW: Birth weight < 1500 g) neonates. Mortality is high, up to 20 to 30%, with the highest rate among neonates requiring surgery [3,4,5,6]. The morbidity of definite (≥Stage II) NEC is significant and includes prolonged hospitalisation, recurrent infections, long-term dependence on parenteral nutrition, and survival with intestinal failure, and long-term NDI, especially in extremely low birth weight (ELBW: Birth weight < 1000 g) neonates needing surgery for the illness [7]. Stey et al. have estimated the cost of treatment of one case of NEC requiring surgery, close to $400,000 [8].

The World Health Organisation (WHO) defines probiotics as “live microorganisms which when administered in adequate amounts confer a health benefit on the host.” [9]. Bacteria such as bifidobacteria, and lactobacilli, and non-pathogenic fungi such as Saccharomyces, are the most common type of microorganisms used as probiotics. The mechanisms for the benefits of probiotics include gut barrier enhancement, immune response modulation (e.g., TLR4 receptor, nuclear factor-B, inflammatory cytokines), and competitive inhibition of gut colonisation by pathogens [10,11]. 

Systematic reviews of randomised controlled trials (RCTs) have shown that prophylactic administration of enteral probiotics significantly reduces the risk of ≥Stage II NEC, mortality, LOS and the time to full enteral feeding in preterm VLBW neonates [12,13]. Many developed countries including Australia, Canada, Finland, Denmark, and Germany, are currently using probiotics as standard practice for prevention of NEC in preterm VLBW neonates [14,15,16,17,18]. A systematic review of observational studies has confirmed the benefits of enteral probiotics in preterm neonates [19]. 

Despite the various systematic reviews of RCTs and ‘before vs. after’ routine use studies (Non-RCTs) reporting significant benefits of probiotics in reducing the risk of ≥Stage II NEC, LOS, all-cause mortality, and feeding intolerance, the uptake of this intervention has been relatively slow, and not universal. The reasons for this slow uptake include the concerns about probiotic sepsis, development and transmission of antibiotic resistance, possibility of an exaggerated pro-inflammatory response, and non-availability of or difficulties in accessing high quality, safe and effective products [20]. In most countries, probiotics are sold as food supplements with poor quality control. The death of a preterm neonate from a contaminated probiotic product that was used in a large RCT by Jacobs et al., has highlighted the issue of poor quality control for probiotic products [21,22]. 

The risk of probiotic translocation and sepsis is higher; especially in critically ill and/or extremely preterm neonates (e.g., suspected/proven sepsis or NEC) with potentially compromised gut integrity [14,23,24,25,26]. Although none of the RCTs have reported probiotic sepsis, there are reports of the administered probiotic causing serious infections such as septicemia, pneumonia and meningitis [24,25,26,27,28,29]. Kopp et al. reported that *L. rhamnosus* GG (LGG) supplementation during pregnancy and early infancy did not reduce the incidence or severity of atopic dermatitis in children, but was associated with an increased rate of recurrent episodes of wheezing bronchitis [30]. It is important to note that probiotic strains may express virulence factors or acquire antibiotic resistance genes via horizontal gene transfer [31]. However, advances in technology have allowed the removal of plasmid for antibiotic resistance from maternal strain [32]. Zheng et al. have emphasised the importance of broader screening of antibiotic resistance in commercially manufactured probiotic supplements. They recommend techniques such as computational simulations, live imaging and functional genomics to study the evolutionary behaviour, adaptations and dynamics of commercially manufactured probiotic for optimising their safety [33]. 

## 2. Para-probiotics

Probiotic products contain a mix of live and dead cells; however, the population of dead cells at any given time during the shelf life of such products is virtually unknown [34,35,36]. The dead cell preparations from probiotics have been fractionated and various cellular components and metabolites have been shown to produce a range of biological responses [34]. Taverniti et al. propose the term ‘paraprobiotic’ (ghost probiotics), to define non-viable microbial cells (intact or broken) or crude cell extracts (i.e., with complex chemical composition), which, when administered (orally or topically) in adequate amounts, confer a benefit on the human or animal consumer [37]. 

Several methods of inactivation of probiotics have been studied including the use of heat, chemicals (e.g., formalin), gamma or ultraviolet rays, and sonication. Different methods of inactivation may affect structural components of the cell differently, and influence its immunomodulatory activity [37]. Heat treatment seems to be the method of choice for inactivation of probiotic strains in majority of studies [37,38].

## 3. Mechanisms of Action of Para-probiotics

The mechanisms of action of heat-inactivated/killed probiotics are poorly understood. A variety of biological responses have been reported after administering killed (mostly heat-killed) probiotics to various mammalian and avian species [37]. Animal studies in gastrostomy-fed infant rats show that live and heat-killed *L. rhamnosus GG* decreases LPS-induced pro-inflammatory mediators and increases anti-inflammatory mediators [39]. Bloise et al. reported similar results on human placental trophoblast cells [40]. Studies in epithelial cells and in an infant formula-fed rodent model suggest that killed microbes may be as effective as live microbes in modulating additional inflammatory stimuli [41,42]. Other potential mechanisms of action of killed/inactivated probiotics include adhesive properties of BoPA a cell surface lipoprotein identified in *Bifidobacterium* (*B.*) *bifidum* and anti-inflammatory effects of *L. acidophilus* and *L. plantarum* [37,43]. Other killed probiotics with beneficial immunomodulatory responses in laboratory settings include *L. rhamnosus GG*, *L. plantarum* L-137, *B. breve*, *Escherichia coli Nissle* 1917, *B. bifidum*, *L. acidophilus*, *L. helveticus*, *B. bifidum* and *L. casei*. [37]. Sakai et al. reported that killed *Enterococcus faecalis* (EC-12) prevented vancomycin-resistant enterococci colonization in the cecum of newly hatched chicks [44]. Reduced capacity for mucosal adhesion is a potential adverse effect of heat inactivation of a probiotic strain. However, contrary to the expectation, strain *P. freundereichii* has been shown to have an increased ability for adhesion after heat inactivation [45]. Reduced ability to exert an anti-inflammatory effect after heat inactivation is another concern. However, heat-inactivated strains *L. casei* strain Shirota or *L. fermentum* MS15 [46] have been shown to modulate inflammatory response by regulating IL-10, human B-defensin and other pro-inflammatory cytokines and *B. breve* and *B. bifidum* have improved ability to increase the secretion of IL-10, an anti-inflammatory cytokine [47]. 

Adams and Kataria et al. have also explored the field of non-viable versus viable probiotic strains. Adams reviewed the ‘probiotic paradox’, and beneficial biological responses of live and dead probiotic bacteria [34]. Enhanced safety and longer shelf life were considered as the advantages of ‘dead’ probiotic bacteria. Dead probiotics had various biological responses including anti-inflammatory effects, attenuation of colitis, reduction of IL-8 production, stimulation of gut immune system, and stimulation of IL-6 production, in pre-clinical studies [34]. Kataria et al. summarised the mechanisms of action of “dead” probiotics or their components, and reported that dead microbes could modulate anti-inflammatory effects as effectively as live probiotics [20].

## 4. Which Probiotic Species Can Be Used in Their Heat-Inactivated form as Para-probiotics?

The *Lactobacillus* and *Bifidobacterium* species are commonly used probiotics. Emerging evidence indicates that strains of both *Lactobacillus* [45,48,49] and *Bifidobacterium* [50,51,52,53,54] species are capable of beneficial effects in their heat-inactivated form. The case of *B. breve* M-16V is worth noting, as this probiotic strain is being used routinely in preterm infants for prevention of NEC [55]. Sugahara et al. have investigated the differences between live and heat-killed *B. breve* M-16V, in the regulation of immune function, intestinal metabolism and intestinal gene expression using gnotobiotic mouse model and omics approaches [50]. Both live and heat-killed forms of *B. breve* M-16V showed immune-modulating effects that suppressed pro-inflammatory cytokine production in spleen cells and affected intestinal metabolism. However, live strains exhibited more significant effects in the regulation of intestinal metabolism and intestinal gene expression involved in nutrient metabolism [50]. Athalye-Jape et al. have reported a strain specific systematic review of RCTs and non-RCTs of *B. breve* M-16V in preterm infants [55]. A total of 5 RCTs (*n* = 482) and 4 non-RCTs (*n* = 2496) were included. Data from the 3 small RCTs (*n* = 386) reporting on clinically important outcomes was inadequate to derive firm conclusions [18,56,57]. Meta-analysis of data from non-RCTs showed significant benefits on LOS, mortality, and the postnatal age at full feeds. There were no *B. breve* M-16V related adverse effects [55]. The findings reported by Sugahara et al., and Athalye-Jape et al. suggest that inactivated *B. breve* M-16V may be a suitable paraprobiotic strain for assessment in clinical trials [50,55]. Research on other paraprobiotics (developed from other probiotic strains that have been shown to be effective in RCTs and/or non-RCTs) is important to study their safety and efficacy against placebo and/or probiotics in preterm VLBW neonates. 

## 5. Clinical and Pre-Clinical Studies of Para-probiotics 

Lahtinen has recently reviewed the pre-clinical and clinical studies on live versus inactivated probiotics [35]. Clinical studies comparing non-viable versus viable probiotic strains for various conditions (e.g., diarrhoea, irritable bowel syndrome, eradication of H. pylori, cow’s milk protein intolerance) were few, and showed comparable, high or lower efficacy of non-viable versus viable probiotics. Small sample sizes, lack of a placebo group, and use of non-standardised strains were some of the limitations of these studies. Findings from various preclinical studies included the following: (1) Viable and non-viable lactobacilli had equal ability for adherence to gut mucosa [58], and heat-killing and protease treatments impaired the mucus adherent property [59]; (2) Heat-killing changed the intestinal location of the bacteria. Live bacteria were seen in Peyer’s patches and lamina propria whereas most heat-killed bacteria were in the lumen and cleared rapidly [60]; (3) Heat-killed lactobacilli inhibited pathogen adhesion to the gut mucosa by competitive exclusion [61]; (4) Inactivated lactobacilli enhanced gut epithelial barrier [62]; (5) Non-viable probiotic components such as cell wall extracts [63], lipoteichoic acid [64], bacterial DNA [65,66], and surface (S)-layer proteins [67] can have immunomodulatory effects by various mechanisms including increased salivary IgA production [68], modulation of host T-cell responses [69] and gene expression [70]; (6) Live and inactivated probiotics had comparable effects on innate immunity [71,72,73]; (7) Live as well as killed *B. lactis* HN019 enhanced phagocytic responses in peripheral blood cells; however, only viable bacteria increased the phagocytic activity of peritoneal cells [74]; (8) As for adaptive immunity, many studies favoured live over non-viable bacteria [60,75,76,77,78], but some showed that both forms had similar effects on the phenotype and functions of human myeloid dendritic cells [79]. Overall, the evidence from pre-clinical and clinical studies suggested that “in some situations, depending on the mechanism of action, probiotic effects are not dependent on cell viability”. The need for clinical studies and consideration of the differences in the effects of dormant (during storage), inactivated and live bacteria was emphasised [80].

## 6. Systematic Review of Studies of Modified Probiotics for Prevention and Treatment of Various Diseases

Zorzela et al. have reported a systematic review of trials of dead bacteria/yeasts (‘Modified microbes’) inactivated by heating/sonication of probiotic strains, for prevention (*n* = 14) or treatment (*n* = 26) of various diseases, mainly in adults and children [38]. The trials compared modified microbes with either placebo (44%) or the same probiotic strain (39%) or standard treatment (17%). Compared with probiotics, the modified microbes were not significantly more or less effective in 86% of prevention and 69% of treatment trials. Meta-analysis of data from 5 RCTs showed significant benefits of modified L. acidophilus (Standard mean difference: −0.81, 95% CI: −1.44, −0.17) as an adjuvant in treatment of acute diarrhoea; however, there was significant heterogeneity (*I*^2^ = 86%). The incidence of adverse events was comparable for modified microbes, probiotics and other controls but many trials did not report adequate data on safety. Overall, there was some evidence that modified microbes may be useful for few conditions. The limitations of this review include the heterogeneity of methodology and the tested strains in the included studies and their small sample sizes [38].

In summary, current evidence indicates that paraprobiotics could be safe alternatives to probiotics in preterm neonates. High quality pre-clinical as well as clinical studies including adequately powered RCTs are warranted in preterm neonates to explore this new frontier. A cluster RCT design is appropriate to avoid the issue of cross-contamination. A non-inferiority design will be acceptable for this purpose. However, deciding the clinically acceptable margin of inferiority will be an important issue considering the effect size for benefits of probiotics for NEC, all-cause mortality, late onset sepsis, and time to full feeding in preterm infants. Considering that effects of probiotic strains are ‘strain-specific’, rigorous assessment of specific effects of different paraprobiotic strains are important. This issue is also relevant when a mixture of strains is used. Assessment of the effects of added prebiotic oligosaccharides is another important issue. Finally, the significance of the utility of paraprobiotics beyond the preterm neonatal population cannot be ignored. 
